# Correlating food and nutritional patterns with cancers in the pediatric oncology population at two specialized hospitals in Tanzania

**DOI:** 10.1186/s40795-024-00824-2

**Published:** 2024-01-11

**Authors:** Dafrosa Joseph Monko, Haikael David Martin, Emmanuel Abraham Mpolya

**Affiliations:** 1 Department of Food and Nutritional Sciences, School of Life Sciences and Bioengineering, Nelson Mandela African Institution of Science and Technology, 447 Arusha, Tengeru Tanzania; 2 Department of Global Health and Bio-Medical Sciences, S chool of Life Sciences and Bioengineering, Nelson Mandela African Institution of Science and Technology, 447 Arusha, Tengeru Tanzania; 3 Bugando Medical Center, Wurzburg Street, 1370 Mwanza, Tanzania; 4 Institute of Bioaffiliationersity, Animal Health & Comparative Medicine, University of Glasgow, G12 8QQ Glasgow, Scotland, UK; 5 Center for Global Health (CGH), Perelman School of Medicine, University of Pennsylvania, 240 John Morgan Bldg, 3620, 19104 Hamilton Walk, Philadelphia, PA USA; 6 Institute for Health Metrics and Evaluation, Population Health Building/Hans Rosling Center, 3980 15th Ave. NE, UW Campus Box #351615, 98195 Seattle, WA USA

**Keywords:** Pediatric oncology population, Clinical nutrition, Nutritional guidelines, Malnutrition, LMIC, Africa

## Abstract

**Background:**

This study of nutritional patterns in relation to cancers among pediatric oncology population in Tanzania was motivated by the lack of up-to-date information about the nutritional practices, the controversy around the importance of nutritional support and the lack of consistent nutritional criteria among pediatric oncology populations.

**Methods:**

A survey study in two cancer referral hospitals of children diagnosed with any cancers, aged between 1 and 17 years inclusive and being eligible for enteral feeding included 131 children. Their demographic, nutritional, feeding and cancer profiles were analyzed descriptively through mapping and other approaches as well as inferentially using multinomial regression models to understand different aspects of nutrition for children suffering from cancers.

**Results:**

The majority (15% or higher) of pediatric oncology population originated from the lake zone. Between 7 and 12% of pediatric oncology population originated from the Western zone. The top-three cancers with their percentages in the brackets were: Wilms Tumor (32%), Acute Lymphoblastic Leukemia (26%) and Retinoblastoma (13%). About 69% of the pediatric oncology population ate foods that are rich in energy but poor in protein such as rice (21.5%), porridge (19.3%), banana (11.7%) and potatoes (10.2%). On the other hand, only 17.5% ate foods that are generally protein-rich such as meat (8.0%), fish (5.3%) and chicken (4.2%); and 12.7% ate milk (4.2%), beans (3.4%), vegetables (2.7%), eggs (1.9%) and fruits (1.5%). Cancers impacted food intake in about 60% of all children with cancers and affected appetite in 18.3% of them. Cancers caused vomiting in 16% and diarrhea in 6.1% of children. The majority of children with cancers (61.8%) took at least one meal while 34.4% took just snacks (*p* < 0.001).

**Conclusions:**

The majority of pediatric oncology population had erratic nutritional patterns and took foods high in energy and poor in proteins. There is a two-way interaction between cancers and nutrition in which cancers affect general nutritional intake which could affect the cancer treatment outcomes in return. Therefore, it is important to consider these interactions while managing pediatric oncology populations in this and similar settings.

**Supplementary Information:**

The online version contains supplementary material available at 10.1186/s40795-024-00824-2.

## Background

Every year, on a global scale, there are about 290,000 new cases of childhood (ages 0–14) cancers and about 100,000 deaths from the same [1, 2]. However, quantification of the true burden of childhood cancers especially from the Low-Middle-Income Countries (LMICs) is hampered by missing data. With these gaps, it is likely that these incidence and mortality statistics are an underestimate [3]. In terms of policy attention, childhood cancers are neglected in the fight against cancers due to several reasons such as their minority in the population of all cancer patients as they are only 1.5% of all incident cancer cases per year [4], poor cultural beliefs of perceiving childhood cancer as deadly and a punishment [5] and finally because more focus is being put on fighting other challenges affecting children such as malnutrition and other diseases [6].

The global burden of pediatric oncology is expected to shift towards Africa by 2050, where almost 50% of the burden will originate [7]. However, understanding of the actual burden in Africa is currently complicated by lack of comprehensive cancer registries [8] as evidenced by a recent review where the share of cancer registries among low-income and low-middle income countries was low, being between 5% and 12% [3], and in some cases none [9]. Other factors making the situation worse in Africa include population growth which leads to an increase in the number of patients, understaffing and generally low prioritization [10–12]. Currently, children with cancers from Sub-Saharan Africa have the lowest survival rates per cancer episode in the world where only 30% or less survive [1, 8]. This is in contrast to high-income countries where up to 80% of children with cancer survive [3].

On top of systemic barriers to proper pediatric oncology care, there are also barriers at the patient level which result into late or missed diagnoses and generally poor cancer care [7] leading to poor pediatric oncology outcomes. Some of the patient/caregiver-level challenges are low knowledge and awareness about pediatric oncology among caregivers, poor socio-economic conditions in general, and treatment abandonment which was reported to be as high as 54% in Kenya [13]. Many reasons for treatment abandonment have been reported including socioeconomic reasons like family poverty, low education, and long travel time to obtain the cancer care. Others include a preference to alternative treatment options, fear of cancer treatments’ side effects, psycho-social factors such as family breakdowns, perceived poor prognosis and reasons falling in domains of provider responses, and other emerging factors as detailed elsewhere [13–16].

The economic factors behind abandonment such as high costs of cancer care are likely to affect the nutritional aspects and lead to malnutrition of the pediatric oncology population due to the opportunity cost of handling the cancer care by foregoing good nutrition. This is likely to lead to malnutrition of these pediatric oncology populations. However, the pediatric oncology population may also have pre-existing malnutrition which is known to be an outcome of a complex interplay of factors and a central determinant of child morbidity and mortality in Africa [17, 18]. Malnutrition in the pediatric oncology population is detrimental due to its association with increased morbidity and mortality [19] from both the malnutrition condition itself and the worsening prognosis of cancer.

Biochemically, cancers in children could cause malnutrition through inflammation during which cytokines released by the tumor alter the metabolism of key biological molecules such as proteins, lipids, and carbohydrates leading to cachexia. Cachexia is a complex metabolic syndrome associated with illness, loss of muscle but without loss of body fat [20]. There are multiple causes of cachexia, including low socioeconomic status, tumor type and stage, host factors, and intensity of treatment, leading to poor nutritional status [21]. However, literature from both clinical and basic research on the impact of nutrition within the pediatric oncology domain remains scarce, so it is important to contribute to this body of knowledge from both observational and experimental research perspectives.

Therapeutically, the use of chemotherapy for treatment of pediatric oncology influences nutrition. A previous study reported an increase in undernutrition by 2% between initiation and during treatment underlining the role of therapy in nutrition [22]. Similar results are echoed by a Turkish study that reported a worsening nutritional status with increasing time spent in chemotherapy among pediatric oncology populations which led to hair loss, pain and lack of energy [23]. These side-effects of chemotherapy, in turn, impact the patient’s optimal nutrient intake [21].

A 2012 systematic review of 46 articles acknowledged the inherent challenges of estimating malnutrition per cancer type but reported that up to 50% of pediatric oncology population presented with malnutrition, the rates differing among cancer types [24]. This systematic review also pointed out gaps in our understanding of the nutritional status and nutritional patterns of different pediatric oncology populations which could be unique in different localities and in different cancer diagnoses.

The current study is aimed at understanding the food and nutritional patterns of pediatric oncology populations at two specialized hospitals in Tanzania. This goal is motivated by the lack of updated information about the nutritional practices among pediatric oncology populations and also the lack of consistent nutritional criteria among the pediatric oncology population in Tanzania [18, 24]. Furthermore, it is motivated by the controversy around the importance of nutritional support among pediatric oncology populations as there is currently no consistency in how to approach it [25]. Filling these gaps will inform efforts to achieve the WHO Global Initiative for Childhood Cancer of ensuring 60% survival for children with cancer aged 0–19 years by 2030 [26].

To that end, this study answers the following five research questions: (i) What is the socio-demographic and oncological profile of pediatric oncology populations in the two specialized hospitals in Tanzania? (ii) What are the common foods taken by such pediatric oncology populations? (iii) What are the nutritional compositions of such common foods and their potential implication in cancer prognosis? (iv) What are the parents’/caregivers’ knowledge and practices in managing the nutrition of children with cancers? And (v), what are the effects of different cancers on the number of meals per day among these pediatric oncology populations?

## Methods

### Design

This was a correlational study using the survey design involving several approaches as follows. Firstly, a cross-sectional survey to collect demographic and nutritional practice information about the pediatric oncology population, secondly, the 24-hour dietary recall, thirdly food frequency survey and finally anthropometric measurements of heights, weights, and mid-upper-arm circumference (MUAC). It also involved retrospective medical records review to collect various clinical information about the pediatric oncology populations.

### Setting

This was a hospital-based study which was undertaken at two cancer specialized referral hospitals in Tanzania, namely, the Muhimbili National Hospital (MNH) and the Bugando Medical Center (BMC). The MNH, located in the commercial capital of Dar es Salaam, is a national referral hospital, research center and university teaching hospital with 1,500 bed capacity and attends up to 2,000 clients per day. The BMC, located in the Lake Zone city of Mwanza, is a 950 + bed capacity tertiary referral hospital which is also one of Tanzania’s largest medical centers and houses a large medical training program. The survey took place from 1st November 2022 to 31st January 2023.

### Participants inclusion and exclusion criteria

The participants for this study were all children diagnosed with cancers and attending or admitted at MNH and BMC within the study period. There were specific inclusion and exclusion criteria as detailed below.

#### Inclusion criteria

The inclusion criteria were all children aged between 1 and 17 years inclusive who had been diagnosed with any type of cancer at any stage and who also had partial or complete gastrointestinal tract function or were eligible for the enteral route feeding.

#### Exclusion criteria

The exclusion criteria were all children aged below 1 years of age regardless of their cancer diagnosis. Also excluded were children aged between 1 and 17 years who were diagnosed with any cancer and had complications which required parenteral feeding.

### Sample size calculations

The sample size calculation was done to have a sample capable of estimating our proportions and other estimate to be within 5% of the true population parameters. In this case, the population is all children diagnosed with cancer and fitting our selection criteria above. The number of children with cancers admitted in the two hospitals is finite and was about 130 by November 2022 (based on a pre-study assessment by the corresponding author (DJM)). This total sample space was the best estimate at the time and could vary in subsequent dates. From this information, the sample size was calculated using the formula by Yamane which is appropriate when the sample space is finite [27]. The formula is given as *n = N/(1 + Ne*^*2*^*)*, where *n* is the sample size we were calculating, *N* is the finite sample space which was 130 and *e* is the margin of error which was taken as 0.05. Calculations gave a minimum sample of 98 participants. During the actual survey a total of 131 participants were accessed, 60 coming from BMC and 71 coming from MNH.

### Sampling

Since participants of the study were hospitalized children with cancers in two tertiary-care hospitals, we reasonably assumed that in terms of the standard of care the pediatric oncology populations in the two hospitals were homogeneous. This eliminated the need for stratification. Therefore, a simple random sampling was adopted in the recruitment of participants. A list of eligible admitted children with cancer was obtained from the hospital administrators. The list of all children meeting the inclusion criteria was made and each child was given an identifying number. Then a random sampling code was written using the R programming language [28] to generate a random number of identifiers to be recruited in the study according to the sample size.

### Data collection tools and variables

The primary data collection tool used in this study was an interview guide on 24-hour dietary recall and food frequency which was adopted from a Kenyan study [29]. A large part of the interview guide was adopted as it was from this Kenyan study. However, some modifications were made to meet our study objectives. Modifications were done on patient information, medical records, and nutritional scores as seen in Supplementary 1. Various sections of the interview guide enabled the collection of socio-demographic information such as age, education, place of origin, marital status and so on. It also enabled the collection of information from medical records such as date of diagnosis and cancer type, type of cancer treatment and any other disease conditions. The interview guides also collected information about the nutritional management and feeding practices of health professionals and caretakers or parents. Furthermore, nutritional information collected was 24-hour dietary recall on the number and types of meals a child was given per day as well as food frequency questionnaire-type questions which queried about the types of food groups eaten per week as well as questions about dietary diversity (See Supplementary 1). We also made use of the Tanzania Food and Nutrition Center (TFNC) food composition Table [30] to understand approximate nutrient compositions of various foods reported.

For consistency in our descriptions, we made use of the definitions of malnutrition according to the World Health Organization (WHO) which involves both undernutrition and overnutrition. Undernutrition is classically subdivided into acute undernutrition (or wasting) -defined by the WHO as weight-for-height (WFH) < -2 standard deviation (SD). On the other hand, chronic undernutrition, (or stunting) -defined as height-for-age (HFA) < -2SD. With overnutrition, age needs to be considered when defining overweight and obesity as follows; for children aged 5–19 years, overweight is defined as BMI (body-mass-index)-for-age > + 1 SD and obesity is defined as BMI-for-age > + 2 SD. Meanwhile, for those under 5 years of age, overweight is defined as WFH > + 2 SD and obesity is defined as WFH > + 3SD [31, 32]. All questionnaires were firstly developed in English and then translated into Swahili language through the recommended steps of forward translation, backward translation, review by an expert committee (HDM and EAM) and finally pilot tested to ensure that no meanings were lost in translation from English to Swahili [33].

### Ethical approval and consent processes

The ethical clearance for this study was granted by the Tanzania Northern Zone Research Ethics Committee, KNCHREC (knchrec.org), with reference number KCHREC/0070/2022. Informed consent for participation was obtained from the legal guardians/parents of children involved in the study. The informed consent process was done as follows: after identification of a child to be included in the study, the researcher (DJM) and research assistant approached the guardian or parent of the child and obtained the informed consent from the parent/guardian. Parents/guardians were allowed to ask questions and were assured that they were free to decline to participate in the study without facing any undue consequences. After obtaining the informed consent the data collection process was done in a way that ensured little disruption of the clinical care.

### Data management and statistical analysis

Data was collected using an open data kit (ODK), stored in a secure server and later downloaded into Microsoft Excel. Data was then cleaned and analyzed using the R statistical analysis software [28]. During data analysis, most variables were analyzed as they were collected but some of the variables were derived. For example, the body-mass-index (BMI) was calculated from the height and weight readings using the formula: *BMI = weight (in kg)/height^2 (in m^2)*. However, BMI is said to be insufficient as the sole means of classifying a person as obese or malnourished due to the involvement of age bands [32, 34]. Therefore, we also measured the mid-upper arm circumference (MUAC) which is a simple tool for screening nutritional status. In classifying nutritional status we used MUAC because it is the better tool for this purpose [35].

Categorical variables were summarized in proportions and chi-squared tests were done to test for the independence of the different levels of variables. Data on the location and proportions of pediatric oncology populations was plotted on the map of Tanzania to get the distribution of childhood cancers in Tanzania among the study population. Information about cancer types and foods was plotted on bar plots arranged from the highest to the lowest for visual and numerical clarity.

Furthermore, foods regularly taken by the pediatric oncology population were plotted against cancer types on heatmaps to visualize the consumption of different foods for different cancers as well as for different days in a 7-day period. A yellow color indicated high intensity while a red color indicated low intensity. White color meant that there was no information provided.

Information from the Tanzania Food Composition Table (30) was used to assess the approximate nutritional value of various foods eaten by pediatric oncology populations in order to obtain an empirical implication of such foods on their general health and also on the implications to the prognosis of the cancers.

Most of the questions had a yes/no answer and sometimes other categories of answers were used such as the number of meals. A chi-squared test was done to test the independence of the levels of two categorical variables. Moreover, the effects of cancers on the number of meals taken by pediatric oncology populations were analyzed using multinomial regression models. For both chi-squared tests and multinomial regressions, results were considered significant when the calculated p-value was < 0.05.

## Results

### Social demographic, income and nutritional profiles of pediatric oncology populations in the two hospitals

The population distribution of sampled children was statistically similar between the two hospitals (BMC and the MNH). Children were divided into three age groups: between 1 and 4 years (or [1-4]), between 5 and 10 years inclusive (or [5–10]) and between 11 and 17 years inclusive (or [11–17]). For clarity, the 5–10 age bracket includes patients up until the age of just before 11 and so on. The age-groups and gender distributions were also statistically similar (Table 1).

**Table 1:** Demographic information of children and their parents

Variable	Levels	n	%	χ^2^ p-value
Hospital				
	Bugando Medical Center (BMC)	60	45.8	0.3365
	Muhimbili National Hospital (MNH)	71	54.2	
Age categories				
	1-4 years or [1-4]	54	41.2	0.04406
	5–10 years or [5–10]	46	35.1	
	11–17 years or [11–17]	31	23.7	
Gender	Female	58	44.3	0.19
	Male	73	55.7	
Place of birth				
	Home	11	8.4	< 0.0001
	Hospital	120	91.6	
Father’s occupation				
	Agriculture	78	59.5	< 0.0001
	Business	18	13.7	
	Professional	24	18.3	
	Technician	6	4.6	
	Unknown	5	3.8	
Mother’s occupation				
	Agriculture	60	45.8	< 0.0001
	Business	27	20.6	
	Housewife	30	22.9	
	Peasant	1	0.8	
	Professional	9	6.9	
	Technician	4	3.1	
Father’s education level				
	Primary	71	54.2	< 0.0001
	Secondary	20	15.3	
	Post-Secondary	14	10.7	
	None	26	19.8	
Mother’s education level				
	Primary	72	55.0	< 0.0001
	Secondary	26	19.8	
	Post-Secondary	9	6.9	
	None	24	18.3	
Marital Status of Parents				
	Married	99	75.6	< 0.0001
	Separated	8	6.1	
	Single	21	16.0	
	Widower	3	2.3	

Most (91.6%) of the sampled children were born in health facilities and majority of their parents (mother and father) were involved in agriculture. Also, most of their parents (both mother and father) predominantly had a primary level of education and were married (75.6%). About 16.0% were single parents (Table 1).

Table 2 shows the income profile of parents and the nutritional status of children in this study. About 10.7% earned below 1 USD while 89.3% earned at least 1 USD per day. About 34% of parents reported earning more than 5 USD per day. In general, the income brackets of most parents and caregivers were below the poverty line of 2.15 USD per day [36]. In terms of the children’s nutritional status, majority (71.0%) were normal, 22.1% were underweight and 6.9% were wasted. Upon inquiring the parents about their opinions on the weight of children since diagnosis, 13% believed that the child weight was constant, while 9.9% felt that the child’s body weight was fluctuating and 6.9% thought that the child’s body weight was poor. Besides, 29.0% said that the child’s body weight was satisfactory, and about 41.2% said that the weight gain speed was slow.

**Table 2:** Income profile of parents and nutritional profile of children

Variable	Levels	n	%	χ^2^ p-value
Reported daily income by parents				
	[0–1) USD	14	10.7	< 0.0001
	[1–2) USD	19	14.5	
	[2–3) USD	19	14.5	
	[3–4) USD	14	10.7	
	[4–5) USD	21	16.0	
	Over 5 USD	44	33.6	
Children nutritional status				
	Normal	93	71.0	< 0.0001
	Underweight	29	22.1	
	Wasting	9	6.9	
Weight gain of child since diagnosis with cancer				
	Constant	17	13.0	< 0.0001
	Fluctuating	13	9.9	
	Poor	9	6.9	
	Satisfactory	38	29.0	
	Slow	54	41.2	

### Distribution of pediatric oncology populations and their locations on the Tanzania map

**Fig. 1:**
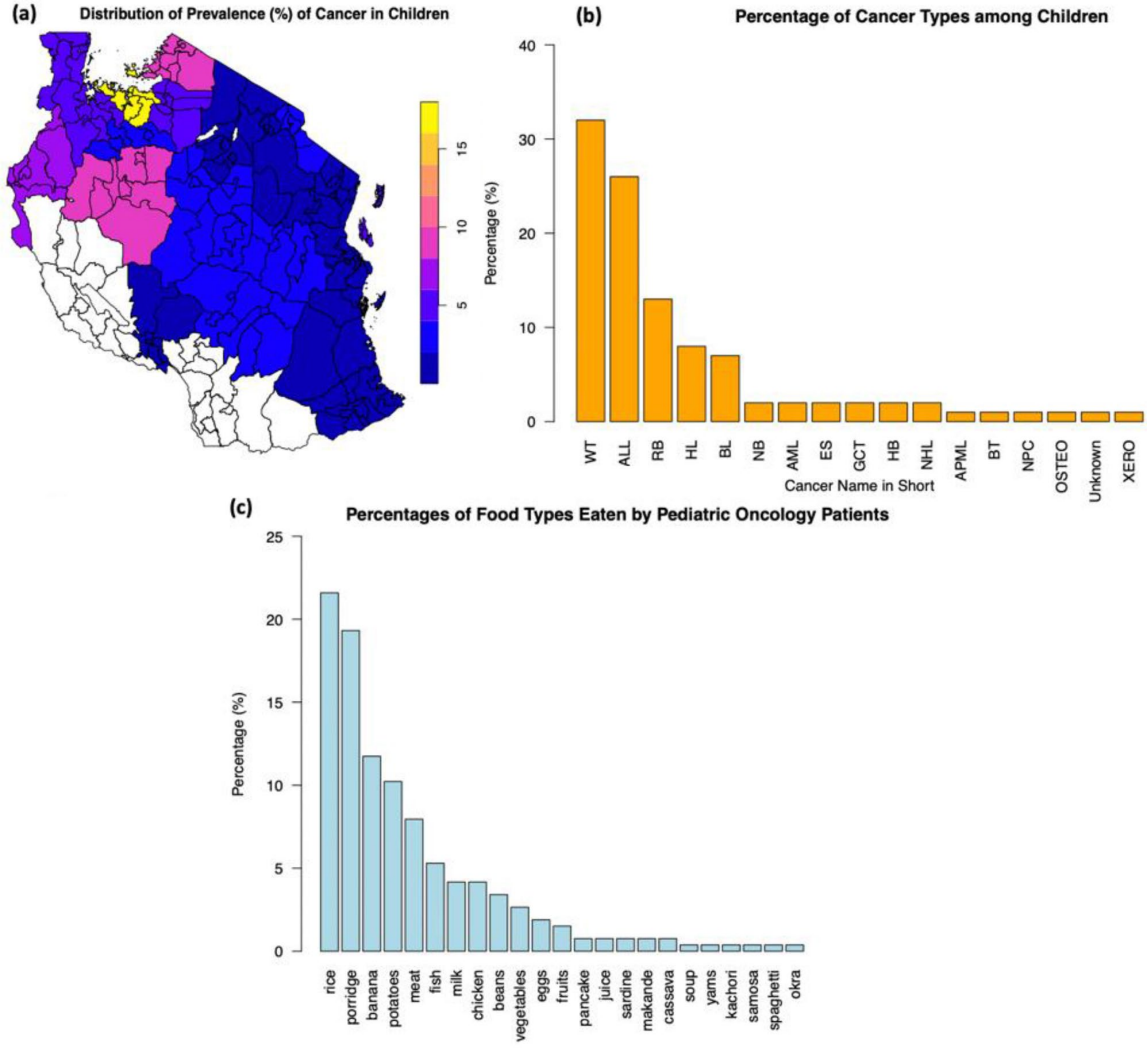
showing different depictions of the study pediatric oncology population. Sub-figure (**a**) is distribution of cancers by areas of origin of the population on the map of Tanzania showing concentrated pediatric oncology in the lake zone and western zone of Tanzania. Sub-figure (**b**) is the percentage of different types of cancers affecting the study population (ALL = Acute Lymphoblastic Leukemia, AML = Acute Myeloid Lymphoma, APML = Acute Promyelocytic Leukemia, BL = Burkitt Lymphoma, BT = Brain Tumor, ES = Ewing Sarcoma, GCT = Germ Cell Tumor, HB = Hepatoblastoma, HL = Hodgkin Lymphoma, NB = Neuroblastoma, NHL = Non-Hodgkin Lymphoma, NPC = Nasopharyngeal Carcinoma, OSTEO = Osteosarcoma, RB = Retinoblastoma, WT = Wilms Tumor and XERO = Xeroderma). Sub-figure (**c**) is the percentage of food types eaten by pediatric oncology population

Figure 1 shows different distribution profiles. Sub-figure (**a**) shows the location of pediatric oncology children on the Tanzania map. The majority of pediatric oncology patients reside in the lake zone where the proportion was 15% or higher (calculated by summing all cancers of the given location divided by the study population). The lake zone in Tanzania comprises the regions which border the Lake Victoria which are Mwanza, Geita, Mara, Kagera, Shinyanga and Simiyu. There are also substantial pediatric oncology patients from the Western zones of Tanzania which include regions of Tabora and Kigoma. Other parts of Tanzania had proportions of less than 2%.

Sub-figure (**b**) shows the percentages of common cancers diagnosed among the studied children. The top-ten cancers with their percentages in the brackets are the Wilms Tumor (32%), Acute Lymphoblastic Leukemia (26%), Retinoblastoma (13%), Hodgkin Lymphoma (8%), Burkitt Lymphoma (7%), Neuroblastoma (2%), Acute Myeloid Lymphoma (2%), Ewing Sarcoma (2%), Germ Cell Tumor (2%) and Hepatoblastoma (2%). Other cancers are also shown in Sub-figure (**b**) but have proportion of 2% or less.

### Common foods taken by pediatric oncology populations

**Fig. 2:**
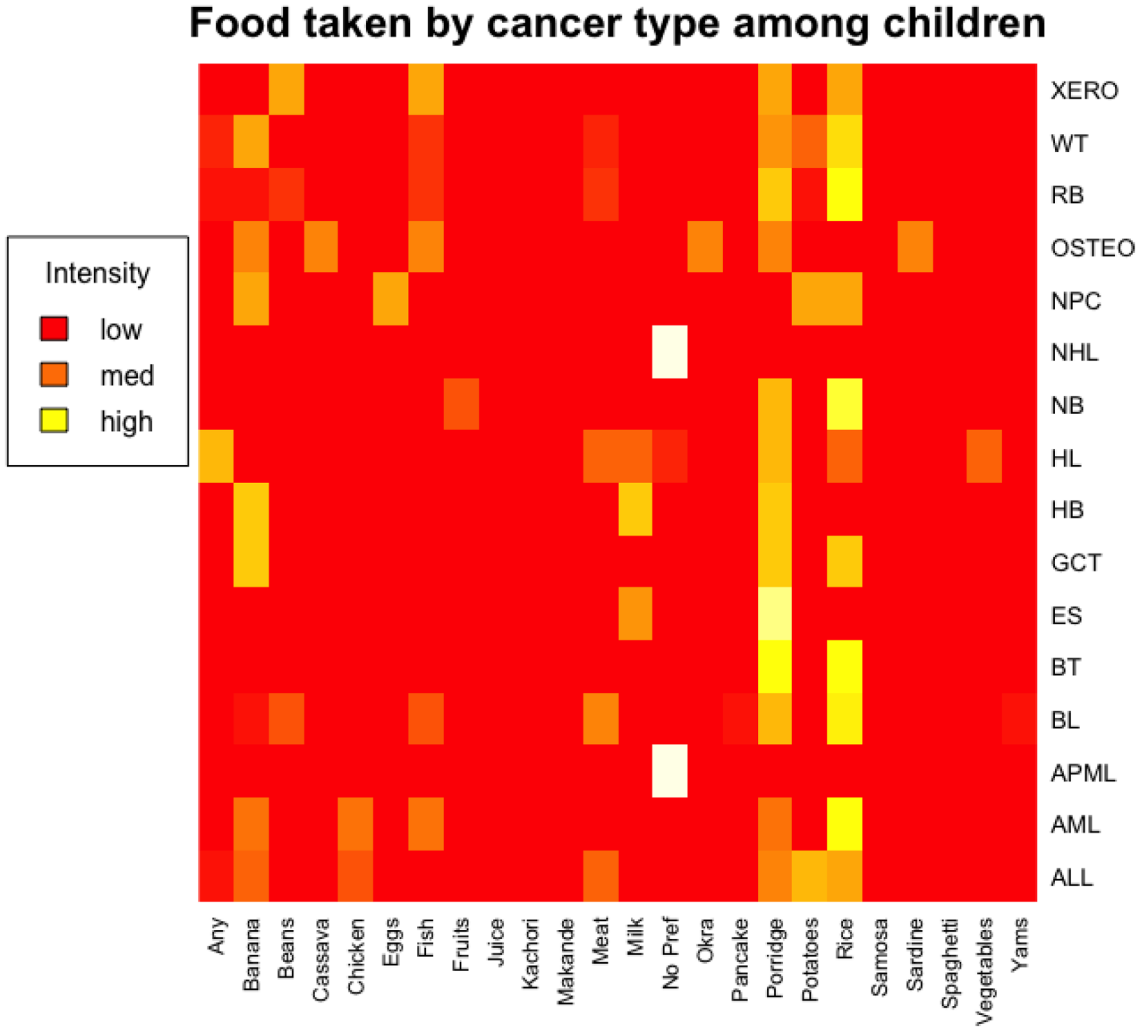
Foods taken by cancer type among the study children. The heatmap ranges from red (low intensity) to yellow (high intensity). White color means missing data values. (ALL = Acute Lymphoblastic Leukemia, AML = Acute Myeloid Lymphoma, APML = Acute Promyelocytic Leukemia, BL = Burkitt Lymphoma, BT = Brain Tumor, ES = Ewing Sarcoma, GCT = Germ Cell Tumor, HB = Hepatoblastoma, HL = Hodgkin Lymphoma, NB = Neuroblastoma, NHL = Non-Hodgkin Lymphoma, NPC = Nasopharyngeal Carcinoma, OSTEO = Osteosarcoma, RB = Retinoblastoma, WT = Wilms Tumor and XERO = Xeroderma)

Figure 1 Sub-figure (**c**) summarizes the types and percentages of foods eaten by the study population over a 7-day period. Foods eaten by at least 10% of studied children were rice (21.5%), porridge (19.3%), banana (11.7%) and potatoes (10.2%). Other foods eaten by less than 10% of the studied children were meat (7.95%), fish (5.3%), milk (4.17%), chicken (4.17%), beans (3.4%), vegetables (2.65%), eggs (1.89%) and fruits (1.52%). Food consumed by less than 1% of pediatric oncology population were juice (0.76%), sardine (0.76%), *makande*–mixture of beans and maize (0.76%), cassava (0.76%), and soup, yams, kachori, samosa, spaghetti and okra (all of which were consumed by only 0.38% of pediatric oncology children). To understand the implication of these foods among pediatric oncology population, we consulted the Tanzania Food Composition Table (FCT) [14] to estimate the nutritional values of the foods eaten. The information extracted from the FCT is shown as Supplementary Table 2 (ST2). In summary most children with cancers in this study consumed energy dense foods with minimal protein, vitamins and minerals sources as further summarized by heatmaps in Fig. 2.

**Fig. 3:**
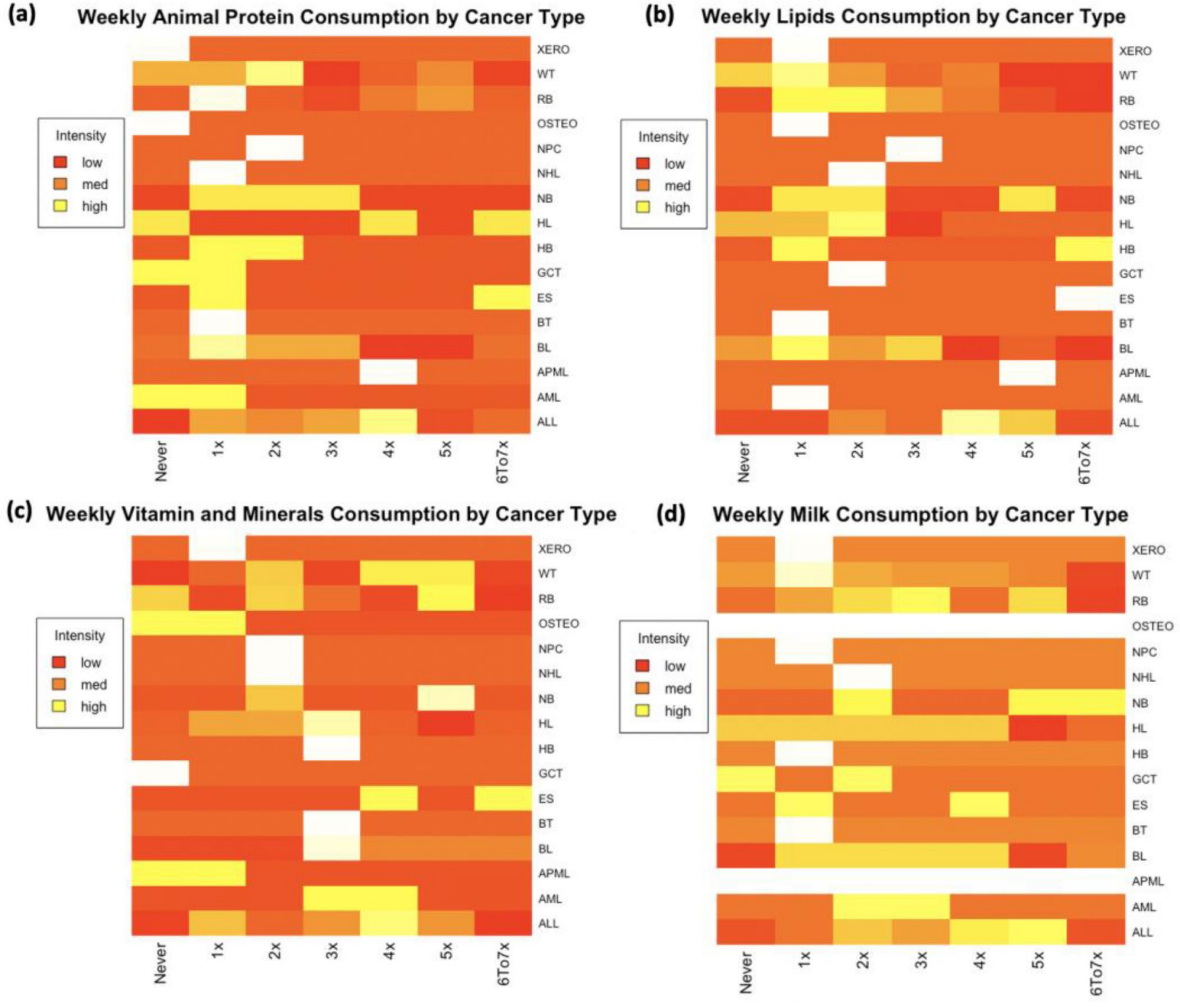
Frequency of weekly intake of different food groups by cancer type. The x-axis is the cancer type and the y-axis is the number of times per week a particular child took a given food. Sub-figure (**a**) is weekly animal protein consumption by caner type. Sub-figure (**b**) is weekly lipid consumption by cancer type. Sub-figure (**c**) is weekly vitamin and minerals consumption by cancer type. Sub-figure (**d**) is weekly milk consumption by cancer type. (ALL = Acute Lymphoblastic Leukemia, AML = Acute Myeloid Lymphoma, APML = Acute Promyelocytic Leukemia, BL = Burkitt Lymphoma, BT = Brain Tumor, ES = Ewing Sarcoma, GCT = Germ Cell Tumor, HB = Hepatoblastoma, HL = Hodgkin Lymphoma, NB = Neuroblastoma, NHL = Non-Hodgkin Lymphoma, NPC = Nasopharyngeal Carcinoma, OSTEO = Osteosarcoma, RB = Retinoblastoma, WT = Wilms Tumor and XERO = Xeroderma)

Figure 3 shows the frequency of daily intake of different food groups by cancer type. From it, we see that there is great variation in daily food intake of pediatric oncology populations per cancer type. For example, with regards to animal proteins (**Sub-figure (a)**), across most cancers, children mainly ate animal proteins only once a week. Across cancer types, some children ate some proteins and some did not eat any during the study period. In only a few cancers, such as neuroblastoma, do we see some actions of eating animal proteins between once up to three times a week. Children with acute lymphoblastic leukemia ate animal protein about four times a week while those with Ewing Sarcoma and Hodgkin Lymphoma ate animal proteins up to 7 times a week (meaning they ate daily). Some children with Hodgkin Lymphoma, Germ Cell Tumor and Acute Myeloid Lymphoma did not eat animal proteins at all during the study period.

Concerning reported daily lipid consumption in a week (**Sub-figure (b)**) there is also a great variation by cancer types. But some patterns can be discerned. For example, the highest rate of consumption of lipid per week was among children with hepatoblastoma (7 times a week) followed by children with neuroblastoma (5 times a week) and acute myeloid lymphoma (4 times a week). In the majority of cancers, children ate lipids only once a week during the study period. It looks like there are a few children with the Wilms tumor who did not eat lipids at all during the study period.

Weekly consumption of vitamins and minerals (**Sub-figure(c)**) also shows great variations with the following key observations. Only children with Ewing Sarcoma ate these micronutrients every day of that week. They were followed by children with Wilm’s Tumor and Retinoblastoma (four to five times a week). Children with acute lymphoblastic leukemia and acute myeloid leukemia ate these micronutrients between three to four times a week). Other than that, the majority of children did not eat enough micronutrients (vitamins and minerals) or ate just once a week.

We also analyzed results for consumption of cow’s milk among pediatric oncology population. Results of cow’s milk consumption are shown in **Sub-figure (d)** and we see that even though some children did not drink milk during the study period, a good number of them had some milk within a week. The majority of children drank cow’s milk between two to five times a week.

### Parents’/caregivers’ knowledge and practices in nutritional management of children with cancers

Supplementary material 1 (ST1) shows results of parents’ or caregivers and health professionals’ (collectively called as caregivers henceforth), knowledge and practice in managing nutritional needs of children with cancers. Starting with a question on knowledge of cancer in the family history, the majority of mothers and/or caregivers (87.8%) reported that they had a history of cancer in their families which implies that they knew something about cancers in general. About 38.9% of mothers and/or caregivers’ reported that cancer always interfered with child’s food intake, while 19.9% said it only interfered sometimes. About 41% of mothers and/or caregivers said that the disease did not interfere with food intake at all.

Caregivers were also asked about the importance of serving foods in small amounts and whether that would also be interfered with by the cancer. We found that 77.1% of caregivers said that when food was served in small amounts, the children were able to eat well compared to when given in large quantities at once. However, concerning appetite, the majority of caregivers (81.7%) said that the cancers did not interfere with the child’s appetite.

Caregivers were also asked whether the cancers caused nausea and only 3.1% said yes. Moreover, 84% of caregivers said that cancer did not cause any vomiting while 93.7% said cancer did not cause diarrhea.

A question was also asked on the number of meals a child took per day. The majority of children took three meals per day (42.7%). About 19.1% of children ate only two meals while 3.8% of children took just one meal per day. There was a substantial percentage of children (34.4%) with cancers who ate just a snack each day. Statistically, these percentages were different among cancer types, meaning that all these percentages occurred partly because of the cancer a child was suffering from. Feeding practices among children with cancers took several forms as follows; about 6.1% of caregivers confessed to using punishment to encourage food intake, while about 22.1% of caregivers said they promoted food intakes through preparation of attractive food.

Given the chronicity of cancer, we also inquired about any lack of efforts to promote food intake due to several factors such as fatigue, lack of time, lack of patience, and having given up on taking care of the pediatric oncology patient. We found that 7.6% of caregivers failed to promote food intake among children with cancer due to lack of time while less than 1% (0.8%) failed to promote food intake due to lack of patience. There were about 3.1% of caregivers who had given up taking care of the pediatric oncology patient and hence failed to promote food intake in their children with cancer.

Another approach to improve a pediatric oncology patient’s food intake is through changing the child’s diet. In this regard, 70.2% of caregivers reported not being able to afford changing the child’s diet while about 7.0% said they would give whatever was available and 1.8% said they would give whatever the child could take. A significant percentage (12.3%) of caregivers said they did not change foods because they were ignorant of the right types of foods.

### Factors affecting the number of meals per day among pediatric oncology populations

To answer this final research question, we performed multinomial regressions to test the effects of each cancer on the number of meals per day (Table 3) The multinomial regression assesses the effects of a predictor variable (a given cancer) on an outcome which has more than two levels (in this case meals per day had four levels: Snacks, One Meal, Two Meals and Three Meals). One of the levels is selected as a reference level (in this case Snacks). A positive coefficient means that the effect of the predictor variable is to increase the chances of that outcome in comparison with the reference level while a negative coefficient decreases the chances of the outcome in comparison to the reference level.

**Table 3:** Multinomial regression for predictors of number of meals per day by cancer type

Predictor variable	Outcome variables (Meals per day)	Coefficient	95% CI [LL, UL]	p-value
Cancer type: Acute myloid lymphoma (AML)	Snacks	Ref		
	One Meal	-0.703	[-0.7028968,-0.7028966]	< 0.001
	Two meals	32.787	[0.097, 0.208]	< 0.001
	Three meals	-14.429	[-15.2562999,-13.6034642]	< 0.001
Cancer type: Brain tumor (BT)	Snacks	Ref		
	One Meal	-2.8869	[-2.8869572,-2.8869572]	< 0.001
	Two meals	-13.362	[-15.3023938,-11.4218131]	< 0.001
	Three meals	-24.2831	[-25.6419594,-22.9243929]	< 0.001
Cancer type: Germ cell tumor (GCT)	Snacks	Ref		
	One Meal	-3.1392	[-3.1392562,-3.1392562]	< 0.001
	Two meals	1.8714	[1.8714274, 1.8714284]	< 0.001
	Three meals	-23.6849	[-26.1490448,-21.2208011]	< 0.001
Cancer type: Neuroblastoma (NB)	Snacks	Ref		
	One Meal	-2.9083	[-2.9083188,-2.9083188]	< 0.001
	Two meals	-12.9757	[-14.6297213,-11.3218508]	< 0.001
	Three meals	0.31374	[-1.0450421, 1.6725328]	0.650867
Cancer type: Non Hodgkin Lymphoma (NHL)	Snacks	Ref		
	One Meal	42.2152	[42.2152541, 42.2152541]	< 0.001
	Two meals	25.84632	[25.8463178, 25.8463194]	< 0.001
	Three meals	-19.6288	[-21.7809622,-17.4768088]	< 0.001
Cancer type: Xerodema (XERO)	Snacks	Ref		
	One Meal	-0.686706	[-1.8296637, 0.4562514]	0.238965
	Two meals	31.20864	[30.2406526, 32.1766393]	< 0.001
	Three meals	-12.61120	[-13.4388368,-11.7835697]	< 0.001
Cancer type: Nasopharyngeal carcinoma (NPC)	Snacks	Ref		
	One Meal	-0.68670	[-1.8762377, 0.5028255]	0.2578574
	Two meals	31.20864	[30.0191143, 32.3981776]	< 0.001
	Three meals	-12.6112	[-12.6112032,-12.6112032]	< 0.001
Cancer type: Hepatoblastoma (HB)	Snacks	Ref		
	One Meal	-0.522057	[-0.5220572,-0.5220572]	< 0.001
	Two meals	-5.142521	[-7.5941707,-2.6908718]	< 0.001
	Three meals	22.06013	[22.0601389, 22.0601389]	< 0.001

Table 3 provides detailed results of the effects of different types of cancers on the number of meals per day. Children with Acute myeloid lymphoma (AML) were more likely to eat two meals compared to snacks (*p* < 0.001) while children with brain tumor (BT) were more likely to eat just snacks (*p* < 0.001).

Among children with germ cell tumor (GCT) they were more likely to eat two meals and a snack than other meal levels (one and three meals) (*p* < 0.001) while children with neuroblastoma (NB) were more likely to eat snacks only (*p* < 0.001). Furthermore, children suffering from Non-Hodgkin Lymphoma (NHL) were more likely to have one or two meals than a snack (*p* < 0.001) while the effects of xeroderma was to favor two meals in comparison with a snack (*p* < 0.001). Children with nasopharyngeal carcinoma were more likely to eat two meals in comparison with the snacks (*p* < 0.001). Finally, children with hepatoblastoma were more likely to have three meals per day (*p* < 0.001).

## Discussion

This study was aimed at describing the food and nutrition aspects of children with cancers in two referral hospitals in Tanzania to increase our understanding of the demands for context-and-cancer-specific nutritional care of pediatric cancer populations. We discuss the profile of the pediatric oncology populations as well as their foods and nutritional aspects before we provide our recommendations, limitations, and conclusions.

### Distribution of pediatric oncology populations and the socio-economic profile of caregivers/parents

Most of the pediatric oncology population came from predominantly agricultural and primary-educated parenthood. These are generally at the lowest income brackets and have some of the lowest awareness about pediatric oncology [12]. They are therefore more likely to associate the disease with witchcraft and thus waste critical time seeking for traditional medical solutions [37]. In addition to negative perceptions about the disease, low education and low socio-economic profile means that the high costs of cancer care lead not only to delayed diagnosis and treatment but also to low survival, which is currently between 5 and 25% in resource-poor countries compared to about 80% in high-resource countries [38, 39].

Plotting the distribution of cancers on the map of Tanzania revealed quite interesting patterns. Majority of pediatric oncology were seen in the lake zone (the area around the Lake Victoria) and western zones. An earlier study which mapped the distribution of pediatric oncology in Tanzania was done by Schroeder et al [40], who showed the count of patients attending the BMC. In the current study we mapped the calculated proportions of cancers derived from the study population for each of the regions in Tanzania. Mapping is important because it brings about an important dimension of visualization and can point to certain unique environmental exposures whenever a clustering of cancer cases is observed. Our presentation, therefore, gives a more specific context of the distributions of pediatric oncology cases in Tanzania. For example, with our findings, more focus must be put on studying various exposures in the Lake and Western zones of Tanzania. However, we appreciate that our mapping did not use inferential methodologies such as those relating the observed pediatric oncology cases to the expected pediatric oncology cases per unit area [41]. We used only the calculated proportion to reveal an alarming pattern of the potential spatial risk distribution of pediatric oncology cases in Tanzania. We call for more application of spatial methodologies (such as small-area methods) to understand the reasons behind distributions of pediatric oncology in Tanzania.

We also reported the most common cancers among our study population. For example, the top-three cancers with their percentages in the brackets are the Wilms Tumor (32%), Acute Lymphoblastic Leukemia (26%), and Retinoblastoma (13%) (Fig. 1b). Nevertheless, a previous study among pediatric oncology populations in northern Tanzania reported the top-three cancers as Burkitt lymphoma, non-Hodgkin lymphoma and Wilms tumor [40]. The difference in the rank between the current study and the study by Schroeder K et al. could be on the area of focus, while they focused on a single specialist hospital, we focused on two specialist hospitals. However, we hypothesize that the differences could also be due to other reasons such as changes in diagnostic technologies and public health awareness that could have caused an increase in detection of new pediatric cancers. Nevertheless, our study updates this previous study and calls for more studies on pediatric oncology in Tanzania in general.

### Foods and nutritional status of children with cancers

With respect to nutritional status, previous studies have reported that in Sub-Saharan Africa about 30% or more of children are malnourished at baseline [42] and this situation only gets worse when suffering from cancers due to metabolically active tumors and starvation as the economically-poor parents/caregivers fail to afford proper food [43]. This study found that about 29% of pediatric oncology population were malnourished. Among them 22.1% were underweight and 6.9% were wasted. This study therefore generally agrees with previous literature. However, we also note that the study group was hospitalized pediatric oncology populations who are already in some form of organized care. The situation in the communities is currently unknown and so we caution any extrapolation of our results to community settings.

### Foods commonly taken by children with cancer in the study population

We report that foods commonly taken by children with cancer in the study population consisted mainly of energy-dense foods with minimal protein, vitamins, and minerals sources. This dietary pattern is not likely to support the growth, development, and healing of these pediatric oncology populations, because being children, they are also in the stage of growth and development. Therefore, the foods must support good health for growth as well as healing. Previous studies have shown that different cancers correlate with malnutrition differently, with lowest proportion of malnutrition (0–10%) among populations with leukemia, relatively high proportions of malnutrition (20–50%) among populations with neuroblastomas and between 0 and 30% for other malignancies [24]. Therefore, proper, and personalized nutritional management of pediatric oncology patients requires an understanding of the underlying cancer of the child.

In Sub-Saharan African countries, most communities rely on one or two staple crops for their daily meals. Readily available in most communities are maize, teff, cassava, yam, sweet potatoes and cooked bananas [44]. These foods are naturally reflected in the foods commonly taken by children with cancers in this study. As much as the uptake might be affected by cancers, as we report, there is a need to further investigate the influence of availability on differential uptakes. A substantial number of children consumed cow milk. There is still controversy around the consumption of cow’s milk vis-a-viz cancers. A previous systematic review reporting mainly on the effects of cow milk among patients reported that it was only beneficial on bone mineral density and reduction of obesity. In this study, cow milk was negatively associated with colorectal, bladder, gastric and breast cancers. However, the general conclusion was that intake of milk and dairy products contributed to meeting nutrient requirements [45].

The consumption of energy-dense foods which we report in this study is not likely to lead to optimal outcomes of cancer treatment due to minimal or no consumption of other food groups. It is not going to meet the dual needs for growth and healing that are needed by these pediatric oncology populations. However, this study asked participants to name the food types they consumed without chemically analyzing the nutritional contents. All information about the nutrient types was obtained through the food composition tables. Therefore, our findings should serve as a platform to further explore the nutritional chemical contents among the foods consumed by the studied pediatric oncology populations. Doing so, will help us move a step closer to more systematic locally-available food-based dietary guidelines just like the previous study by Du Plessis et al [46], but with more emphasis among pediatric oncology populations to address their individualized needs based on their specific cancers.

Besides, in this study, we found that children with different types of cancers took different types of foods along such a wide spectrum that it is difficult to draw strong conclusions. Existing research report that pediatric oncology populations are more likely to exhibit unhealthy dietary behaviors [47] because of several factors related to the cancer itself, the treatments involved, and the psychosocial impact of the disease [21]. Other factors could be dietary restrictions, emotional distress and lack of nutritional support in general [13, 15]. Given the wide spectrum of factors that can affect the nutritional intake [48, 49], this study calls for more studies to define a personalized and context-specific nutritional care of children sufferin gfrom cancers.

### Parents’/caregivers’ knowledge and practices in managing the nutrition of children with cancers.

In general, more than 50% of parents’/caregiver’s had an opinion that cancers interfered with food intake of their children and also stated that when food was served in small amounts, children were able to eat well compared to when given in large quantities at once. This result means that caregivers/parents have some understanding of ways to improve the food intake of their children However, a large number (87.8%) of parents or caregivers had limited knowledge about nutrition of children with cancers, a result which has been echoed by other commentators such as Montgomery et al [50]. This calls for enhancement of the nutritional knowledge and practice among caregivers and parents of children with cancers in this and similar settings.

We also found a great variation in the feeding patterns of children with cancers in terms of the number of meals taken per day. For example, we found that children with Acute myeloid lymphoma (AML) were more likely to eat two meals compared to snacks while children with brain tumor (BT) were more likely to eat just snacks. We also found that there was a substantial percentage of children (34.4%) with cancers who ate just a snack each day and finally that neuroblastoma (NB) was more likely to eat snacks only. Previous studies on adults have tried to quantify the role of number of meals on cancer outcomes [51], but there are no such studies among pediatric oncology populations. Our study therefore serves as one of the first to attempt to quantify the association between a cancer type and the number of meals. Our findings should therefore be used as a baseline for further studies using more sophisticated designs and analytical approaches to understand these associations.

Nutrition for children is based on the same principles as nutrition in adults, however, children need different amounts of specific nutrients at different ages because they also must grow. Most studies on nutrition in relation to cancers are performed on adults and thus the role of diet in childhood cancer is less well understood [52]. The great variation in food intake which this study reports reflects this vague understanding of the role of foods in childhood cancers and the role of childhood cancers in food intake. Therefore, we reiterate our call for more studies in this area so that more context-specific nutritional guidelines can be developed for pediatric oncology populations.

## Conclusions

This study on food and nutritional patterns in relation to cancers among pediatric oncology populations at two specialized hospitals in Tanzania revealed a higher proportion of childhood cancer within the lake zone of Tanzania, where the leading cancer type is the Wilms tumor. Most parents had only a primary educational status and were predominantly agriculturists, both being recipes for potential poverty and malnutrition of their children. There was a troubling pattern of food intake among the study children due to limited nutritional care knowledge among the parents/caretakers, unsystematic feeding styles and potentially the cancer types suffered by children. A preponderance of energy-dense foods, and meager proteins and other micronutrients intake would not meet the growth and healing needs of these children. We also found potential correlations between cancer types and the frequency of meals taken by the pediatric oncology populations which is important if we are to individualize the nutritional care of children for each cancer type.

### Recommendations

From our findings we recommend that a comprehensive clinical nutritional assessment should be part and parcel of pediatric oncology care and should be continuous. There is also a need to develop context-specific nutritional guidelines to support quality nutritional care of children with cancers through simple and understandable pieces of information. We suggest that each child suffering from any cancer must receive a more individualized focus when managing their nutrition in relation to the disease. Furthermore, we recommend a comprehensive nutritional education package for parents and caretakers to improve the understanding of cancers, their treatments, and their effects on the nutritional behavior of the children with cancers. Finally we recommend further studies to understand some of the correlations found in this study such as reasons for spatial distribution of pediatric oncology populations in Tanzania, reasons for intake of different foods by cancer type, reasons for different frequencies of food intake by cancer types and finally the possibility or challenges of personalized clinical nutritional care in resource-poor settings and how to develop context-specific nutritional guidelines which will make good use of available foods for optimal nutritional benefits.

### Study limitations and further research

Finally, our study was not without limitations; this being a correlational study, our findings could only be indicative of a potential pattern, not a causal relationship between an exposure and an outcome. To establish causal pathways, a follow-up study would be more appropriate. We also note that another confounder is the fact that there are many types of brain tumors, some of which are associated with diencephalic syndrome which is characterized by profound emaciation and failure to thrive with normal caloric intake and normal linear growth. Also, our age grouping into 1 to 4 years, between 5 and 10 years and between 11 and 17 years is deviating a bit from the standard groupings [53] which is toddler to early childhood (between 13 months and 5 years), middle childhood (between 6 and 11 years) and early childhood (between 12 and 18 years). Currently, we do not have evidence that this grouping can lead to different conclusions, but we mention it here as a potential area for improvements in the future. Given the wide spectrum of factors which can cause differential food intakes, we avoid making strong conclusions but call for more future research to understand the following areas: (i) Research on community-based cancer care among children with cancers to enrich our knowledge around this area because the current study was only hospital-based; (ii) Research on understanding the role of economic, psychosocial, biochemical, and physiological predictors of intake of different foods among pediatric oncology populations. (iii) Research on assessing the reliability of parents’ recall of variables belonging to their children such as weight change of their children in follow-up designs. And, (iv) Research on similar predictors but among children aged less than 1 year old as these were excluded in the current study. Despite these limitations, the current study’s has exposed a chaotic food consumption pattern among pediatric oncology populations in two referral hospital in Tanzania and brings to the attention of clinicians and policy makers about the need to strengthen the nutritional management of pediatric oncology populations and develop context-specific nutritional guidelines for them.

### Electronic supplementary material

Below is the link to the electronic supplementary material.

**Supplementary material:** Questionnaire, chi-squared tests and food composition table

## Data Availability

The datasets generated and/or analysed during the current study are available in the GitHub repository, https://github.com/em1601/dafmo_data?search=1.

## List of abbreviations

ALL: Acute Lymphoblastic leukemia
AML: Acute Myeloid Lymphoma
APML: Acute Promyelocytic Leukemia
BL: Burkitt Lymphoma
BMC: Bugando Medical Center
BMI: Body-Mass Index
BT: Brain tumor
DJM: Dafrosa Joseph Monko
FBDGs: Food-based dietary guidelines
ES: Ewing Sarcoma
FCT: Food Composition Table
GCT: Germ Cell Tumor
HB: Hepatoblastoma
KCHREC: Northern Zone Research Ethics Committee
MNH: Muhimbili National Hospital
MUAC: Mid-upper-arm circumference
NB: Neuroblastoma
NHL: Non-Hodgkin Lymphoma
NPC: Nasopharyngeal Carcinoma
ODK: Open Data Kit
OSTEO: Osteosarcoma
RB: Retinoblastoma
TFNC: Tanzania Food and Nutrition Center
USD: US Dollars
WHO: World Health Organization
WT: Wilms Tumor
XERO: Xeroderma
