# Supplementary material for: Correlating food and nutritional patterns with cancers in the pediatric oncology population at two specialized hospitals in Tanzania
Source: BMC Nutr. 2024 Jan 11;10:10. doi: 10.1186/s40795-024-00824-2 (PMC10782733; doi:10.1186/s40795-024-00824-2)
Supplement: Supplementary file 1 — Supplementary material: Questionnaire, chi-squared tests and food composition table [file 40795_2024_824_MOESM1_ESM.docx]

Supplementary information

**Supplementary 1: Modified Questionnaire**


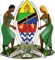
NELSON MANDELA AFRICAN INSTITUTION OF SCIENCE AND TECHNOLOGY (NM-AIST)
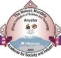


INFORMED CONSERT FORM

To assess Nutrition Management Knowledge, and Practices (KP)among Health Providers/Professional and Parents, (Father, mother) OR Caregivers of Children with Cancers Tanzania

INTRODUCTION TO PARTICIPANT

Take time to greet the Respondent. Hello……. My Name is………………………………. from……………………………

Thank you again for dedicating time to speak with us today. The aim of this research is to Gather the information from you which will help to Formulate Nutrition Guidelines for Management of Children with Cancer Before, During and After their Treatment.

We are visiting here to ask more information about what are the Practices and Attitudes during feeding the children diagnosed with Cancer? There are no right or wrong answers. We are asking to better understand what children Eats and Managed, please feel free not to answer any question that you are not comfortable with. There is no penalty to you for not answering a question you might not be comfortable with.

Name………………………Parent……………...Care takers……………. Sex……………

Date ………………. Month………………. Year…………………. Signature………. Or Finger Print…………….

Thank You

DOCUMENT NO. 1;

**1; RESEARCHER ADMINISTERED QUESTIONNAIRE**

Administrative details Questionnaire Code NO. …………………

Name of the interviewer ………………Code No. ……Date of interview ………………

Time started interview……………… Time finished interview……….

SOCIO-ECONOMIC STATUS; 1. Age… a) below 5 years b) 5-10 years c)11-17 years

2. Gender: male ………Female ……… 3. Place of birth of child: Home… Name-

Hospital….……… 4. Date of birth of child……………., 5. Tribe… ,,,,,,,,,,,…

6. District/Residence…………7. Region…….................8. Nationality………………

9. Total number of siblings …………… 10. Fathers/Mothers main Occupation 11.

Age………….12. Education level of Father/Mother 13. Care giver(s)

Occupation …………. …………. ………14. Marital status of parents…………….

1. What is your family’s total monthly income (in TZ. shs)? a. 0 – 2000 b. 2001 – 4000 c. 4001 – 6000 d.6000 – 8000 e. 80001 – 10000 f. over 10,000
2. Do you have any other source of income/livelihood? a. yes b. no
3. If yes which source a) Crop income b) Livestock income c) Both the above

**CHILD ANTHROPOMETRY/NUTRITIONAL STATUS**

- 1. Anthropometric measurements 1st reading 2nd reading average

Weight (Kg) …………… …………… ………… Height/length (cm) ……………

Then compute the following: BMI (>5 years) -------------------- BMI/Age----------------

Weight/Age (underweight)----------------- Height/Age (stunting) -----------------

Weight/Height (wasting)---------Normal……………….

- 1. How has been the weight gain since diagnosis? (Child nutritional status)
     1. Poor
     2. Fluctuating
     3. Slow
     4. Satisfactory
     5. Constant

**NUTRITIONAL MANAGEMENT PRACTICES**

1. Is there history of cancer in your family?
   1. Yes (specify who)
   2. No
   3. Don’t know

1. Does the disease state/treatment given interfere with the child food intake?
   1. Yes
   2. No
   3. Sometimes
   4. Don’t know

1. Does the disease state/treatment given interfere with the child food intake?
   1. Yes
   2. No
   3. Sometimes

1. If yes or sometimes, how does it interfere with the child’s intake of food
2. Eat very little food
3. Appetite problems
4. Has nausea
5. Vomiting
6. Diarrhea
7. Any other specify………………….
8. If b above, how does you describe your child appetite now or most of the time? a. good
9. Moderate
10. Poor
11. Very poor

1. How many meals does your child take in a day?
   1. One
   2. Two
   3. Three
   4. More than 3 + snacks
   5. Snacks only

1. In case of poor appetite is your child assisted to eat in hospital or at home
   1. Yes
   2. No
   3. Sometimes

1. (A) Are you encouraged to eat when your appetite is poor? a. Yes, b. No (B) If yes how do you do it? a. Use a stick b. Prepare attractive food c. Serve small amount of food d. Others (specify)… (C) If no, why? a. Lack of time

b. Lack of patience c. Given up d. Specify others……………

1. (A). What attempts are being made by the hospital to improve your food intake?
   1. Give multivitamin to boost appetite
   2. Nutrition education
   3. Enteral /parenteral nutrition
   4. Nothing

(B) Have they been successful? a. Yes b. No

**METABOLIC DEMAND**

1. Metabolic stress determined by number of variables known to cause protein& calorie needs

Stress ……. none (0) low (1) moderate (2) ……high (3)

- Fever
- Fever duration
- Use of drugs
- None

**FOOD CONSUMPTION**

1. List down the foods you like and those u disliked Food liked Foods Disliked

…………………………. ………………………. …………………………..

1. (a) Do you crave for any particular foods? a. Yes b. No (b). If yes list some of

them ……………………………………………………………………………………

3.(a). Are there foods that your child does not eat completely? a. Yes b. No (b) If yes which are some of these foods…………………………

1. (a) Have you been told to change your child’s diet since the doctor learnt

he/she had cancer? a. Yes b. No (b) If yes what was the reason given?

…………………………………………………. (c) Have you yourself changed the

diet? a. Yes b. No (d) If no, why?

…………………………………………………………………………

1. If yes how does your child follow the prescribed diet? a. Strict adherence b. Rarely c. Sometimes d. Never
2. Do you agree that these foods are best for your Childs disease state? a. Yes b. No

1. What are some of the constraints you face in proper dietary planning for your child?
   1. Lack of time
   2. Lack of patience
   3. Given up

d. Specify others…………………………………………………………………

1. Please estimate the average food and the amount of it eaten per week by Specifying type of food & Cooking methods used for Last 7 days, with/without sugar/salt added

1. Carbohydrates; rice, maize, wheat, sweet potatoes, Irish potatoes, yams, gimbi, cassava, finger millet, sorghum and Others specify Example; Boiled sweet

potatoes ONCE A WEEK

- 1. Never
  2. Once a week
  3. 2 times a week
  4. 3 times a week
  5. 4 times a day
  6. 5 times a week
  7. 6-7 a week

1. Protein Animal source;

Beef, goat, sheep, chicken, egg, fish, sardine, senene, /Other specify…. (E.g., Fried meat,

1pc roasted chicken/boiled fish medium size)

- 1. Never
  2. Once a week
  3. 2 times a week
  4. 3 times a week
  5. 4 times a day
  6. 5 times a week
  7. 6-7 a week Plant source;

Legumes Lentils/beans; black bean, soya beans, peas, mixed beans….

1. Never
2. Once a week
3. 2 times a week
4. 3 times a week
5. 4 times a day
6. 5 times a week
7. 6-7 a week

1. Lipids

Sunflower oil, groundnut oil, olive oil, palm oil, sesame, pumpkin seed oil,

Butter oil, others specify……

- 1. Never
  2. Once a week
  3. 2 times a week
  4. 3 times a week
  5. 4 times a day
  6. 5 times a week
  7. 6-7 a week

1. Milk and milk product
   1. Never
   2. Once a week
   3. 2 times a week
   4. 3 times a week
   5. 4 times a day
   6. 5 times a week
   7. 6-7 a week
2. Vitamins & minerals,

Vegetables & fruits specify list…….

- 1. Never
  2. Once a week
  3. 2 times a week
  4. 3 times a week
  5. 4 times a day
  6. 5 times a week
  7. 6-7 a week

1. Spicy & condiments; List/Write down type of spicy and the mount
   1. Never
   2. Once a week
   3. 2 times a week
   4. 3 times a week
   5. 4 times a day
   6. 5 times a week
   7. 6-7 a week
2. Water; (E.g., 2 glass every day=14glasses per week)
   1. Never
   2. Once a week
   3. 2 times a week
   4. 3 times a week
   5. 4 times a day
   6. 5 times a week
   7. 6-7 a week

1. Juice and other fluids

Fruit juices, homemade/Industrial, local -made outside home

- 1. Never
  2. Once a week
  3. 2 times a week
  4. 3 times a week
  5. 4 times a day
  6. 5 times a week
  7. 6-7 a week

1. Lipids

Example; Sunflower oil, Groundnut oil, olive oil, palm oil, sesame, pumpkin seed oil,

Butter oil, others specify……

- 1. Never
  2. Once a week
  3. 2 times a week
  4. 3 times a week
  5. 4 times a day
  6. 5 times a week
  7. 6-7 a week

k. Others special food specify……………….

1. Never
2. Once a week
3. 2 times a week
4. 3 times a week
5. 4 times a day
6. 5 times a week
7. 6-7 a week

………………………………………END……………………………………

**2; OBSERVATION CHECKLIST**

Yes ……. No ……Sometimes ………

1. Is the patient able to sit upright? ……..…. ……….. ………….

Is the patient able to walk? ……..…. ……….. ………….

Is the patient totally incapacitated? ……..…. ……….. ………….

Can patient eat food without assistance? ……..…. ……….. ………….

1. General conditions of the child a) child is dull b) active c) disinterested

1. Clinical signs of nutritional status 1 2 3 4

Skin -Rashes rough skin sores normal

Hair -Sparse hair silky hair brown hair black hair

Teeth -White brown molted dental carries

Mouth- Sore mouth dry mouth unable to chew normal

Eyes- Red eyes swollen eyes itchy eyes normal

1. Body Fluid status;

- Ankle edema
- Ascites
- Lower limb
- Upper limb
- Face
- No edema
- Another place, specify ……………

**3; MEDICAL HISTORY FORM (Filled from hospital records) Code No: …**

1.Date of diagnosis………………

1. On diagnosis of disease

Weight……………………………………………………….………………….

Height/length……………………………………………….……………………

MUAC……………………………………………………………….

BMI………………………………………………….……………………….

1. Date Child started on treatment …………...
2. Treatment method given a) chemotherapy and or b) radiotherapy, Surgical

with chemotherapy, immunotherapy……… other specify………...

1. Date of last visit to hospital/center ………………
2. Anthropometric measurements (1) in the last visit
   1. Weight /Age (Underweight)…………………………………………

Height / Age (Stunting)

Height or Length /Height (Wasting)………………………

BMI for age (>5yrs old) ……………………………………………….………………

- 1. Anthropometric measurements (2) in the last visit

MUAC (mid-upper Arm Circumference) …………………………………….

- 1. Anthropometric measurements (3) in the last visit

Abdominal circumference (AC)………………………………………

1. Which cancer does the child suffer from a. Acute Lymphoblastic Leukemia b. Kidney

cancer c. Hodgkin’s disease d. Burkett’s Lymphoma e. Ewing sarcoma f. Retinoblastoma

g. Any other specify …………………

1. Stage of cancer during first visit to hospital a. stage one of cancer

b. Stage 2 cancer, Stage 3 of cancer, Stage four, End stag of life Palliative stage 8. Major complications/identified …………………………., ,

9.. Any Identified nutritional problem(s) from records ……………………

1. PRIMARY/ secondary nutritional biomarkers recorded in file/ from laboratory examination results
2. Is there any surgical procedure done following cancer problems?...........................
3. Is there any surgical procedure done apart from cancer……a. Nes, b. no, c, Not Known.

**4: NUTRITIONAL RISK SCORE (NRS-2002)**

CODE NO/ TITTLE of Assessor. …………

- 1. PATIENT WEIGHT ………………….
     - 100- Expected weight for length
- 90 – 99% Of expected weight for length 1
- 80 – 89% Of expected weight for length 2
- ≤ 79% Of expected weight for length 3
  1. APPETITE
     - Good appetite manages most of 3 meals/day 0
     - Poor appetite, poor intake 1
     - Appetite nil unable to eat 2

- 1. ABILITY TO EAT/RETAIN FOOD
     - No difficulties in eating Able to eat independently 0
- Problem holding food 1
- Difficulty swallowing regains modified consistency 2
- Unable to take food orally, severe vomiting and diarrhea 3
  1. . STRESS FACTOR
     - No stress 0
     - Mild minor surgery 1
     - Moderate chronic disease major surgery, infections 2
     - Severe multiple injuries, carcinoma/malignant diseases 3
  2. Expressing their experienced quality of life
     - 100 – fully active, normal
     - 90 – minor restrictions in strenuous physical activity
     - 80 – active, but tired more quickly
     - 70 – greater restriction of play and less time spent in play activity
     - 60 – up and around, but active play minimal; keeps busy by being involved in quieter activities 50 – lying around much of the day, but gets dressed; no active playing participates in all quiet play and activities
     - 40 – mainly in bed; participates in quiet activities
     - 30 – bedbound; needing assistance even for quiet play
     - 20 – sleeping often; play entirely limited to very passive activities
     - 10 – doesn’t play; does not get out of bed
     - 0 – unresponsive

- 1. SYMPTOMS: I had the following problems that have kept me from eating enough during past two weeks (child presented with.)
- NO problems eating
- No appetite…
- Nausea &vomiting
- Constipation
- Mouth sores
- Things taste funny/no taste
- Problems swallowing
- Pain; where…….
- Diarrhea
- Dry mouth…., Smell bother……Fill full quickly……
- Fatigue…………, Other… e.g., depression, money, dental problem

……………SCORES TOTAL SCORE

**Supplementary Table 1 (ST1):** Children cancer profile and caregiver/health professional feeding knowledge and practices

| **Variable** | **Levels** | **n** | **%** | **χ^2^ p-value** |
| --- | --- | --- | --- | --- |
| Presence of cancer history in the family |  |  |  |  |
|  | Yes | 115 | 87.8 | < 0.0001 |
|  | No | 8 | 6.1 |  |
|  | Unknown | 8 | 6.1 |  |
|  |  |  |  |  |
| Whether cancer disease interferes with child’s food intake |  |  |  |  |
|  | Yes | 51 | 38.9 | 0.004462 |
|  | No | 54 | 41.2 |  |
|  | Sometimes | 26 | 19.9 |  |
|  |  |  |  |  |
| Whether it interferes with intake of little food |  |  |  |  |
|  | Yes | 30 | 22.9 | < 0.0001 |
|  | No | 101 | 77.1 |  |
|  |  |  |  |  |
| Whether it interferes with appetite |  |  |  |  |
|  | Yes | 24 | 18.3 | < 0.0001 |
|  | No | 107 | 81.7 |  |
|  |  |  |  |  |
| Whether it caused nausea |  |  |  |  |
|  | Yes | 4 | 3.1 | < 0.0001 |
|  | No | 127 | 96.9 |  |
|  |  |  |  |  |
| Whether it caused vomiting |  |  |  |  |
|  | Yes | 21 | 16 | < 0.0001 |
|  | No | 110 | 84 |  |
|  |  |  |  |  |
| Whether it caused diarrhea |  |  |  |  |
|  | Yes | 8 | 6.1 | < 0.0001 |
|  | No | 123 | 93.7 |  |
|  |  |  |  |  |
| Meals taken by child |  |  |  |  |
|  | One Meal | 5 | 3.8 | < 0.0001 |
|  | Two Meals | 25 | 19.1 |  |
|  | Three Meals | 56 | 42.7 |  |
|  | Snacks Only | 45 | 34.4 |  |
|  |  |  |  |  |
| Promoting food intake by punishment |  |  |  |  |
|  | Yes | 8 | 6.1 | < 0.0001 |
|  | No | 123 | 93.9 |  |
|  |  |  |  |  |
| Promoting food intake by preparing attractive food |  |  |  |  |
|  | Yes | 29 | 22.1 | < 0.0001 |
|  | No | 102 | 77.9 |  |
|  |  |  |  |  |
| Promoting food intake by feeding in small amounts |  |  |  |  |
|  | Yes | 42 | 32.1 | < 0.0001 |
|  | No | 89 | 67.9 |  |
|  |  |  |  |  |
| Lack of effort to promote food intake due to lack of time |  |  |  |  |
|  | Yes | 10 | 7.6 | < 0.0001 |
|  | No | 121 | 92.4 |  |
|  |  |  |  |  |
| Lack of effort to promote food intake due to lack of patience |  |  |  |  |
|  | Yes | 1 | 0.8 | < 0.0001 |
|  | No | 130 | 99.2 |  |
|  |  |  |  |  |
| Lack of efforts to promote food intake because given up |  |  |  |  |
|  | Yes | 4 | 3.1 | < 0.0001 |
|  | No | 127 | 96.9 |  |
|  |  |  |  |  |
| Attempts made by hospital to boost food intake: multivitamins |  |  |  |  |
|  | Yes | 15 | 11.5 | < 0.0001 |
|  | No | 116 | 88.5 |  |
|  |  |  |  |  |
| Attempts by hospital to boost food intake: nutritional education |  |  |  |  |
|  | Yes | 37 | 28.2 | < 0.0001 |
|  | No | 94 | 71.8 |  |
|  |  |  |  |  |
| Have the measures been successful? |  |  |  |  |
|  | Yes | 94 | 71.8 | < 0.0001 |
|  | No | 37 | 28.2 |  |
|  |  |  |  |  |
| Reasons for not being able to change child’s diet |  |  |  |  |
|  | Can’t afford | 40 | 70.2 | < 0.0001 |
|  | I give child what is available | 4 | 7.0 |  |
|  | I give what the child likes | 1 | 1.8 |  |
|  | I am ignorant to the right food | 7 | 12.3 |  |
|  | Food is unavailable | 5 | 8.8 |  |
|  |  |  |  |  |
| Feeding child food without salt or sugar once a week |  |  |  |  |
|  | Yes | 18 | 13.7 | < 0.0001 |
|  | No | 113 | 86.3 |  |
|  |  |  |  |  |
| Feeding child food without salt or sugar twice a week |  |  |  |  |
|  | Yes | 9 | 6.9 | < 0.0001 |
|  | No | 122 | 93.1 |  |
|  |  |  |  |  |
| Feeding child without salt or sugar three times a week |  |  |  |  |
|  | Yes | 12 | 9.2 | < 0.0001 |
|  | No | 119 | 90.8 |  |
|  |  |  |  |  |
| Feeding child without salt or sugar four times a week |  |  |  |  |
|  | Yes | 12 | 9.2 | < 0.0001 |
|  | No | 119 | 90.8 |  |
|  |  |  |  |  |
| Feeding child without salt or sugar five times a week |  |  |  |  |
|  | Yes | 8 | 6.1 | < 0.0001 |
|  | No | 123 | 93.9 |  |
|  |  |  |  |  |
| Feeding child without salt or sugar five six to seven times a week |  |  |  |  |
|  | Yes | 38 | 29 | < 0.0001 |
|  | No | 93 | 71 |  |
|  |  |  |  |  |
| Never fed child protein animal source |  |  |  |  |
|  | Yes | 16 | 12.2 | < 0.0001 |
|  | No | 115 | 87.8 |  |
|  |  |  |  |  |
| Protein animal source once a week |  |  |  |  |
|  | Yes | 35 | 26.7 | < 0.0001 |
|  | No | 96 | 73.3 |  |
|  |  |  |  |  |
| Protein animal source twice a week |  |  |  |  |
|  | Yes | 22 | 16.8 | < 0.0001 |
|  | No | 109 | 83.2 |  |
|  |  |  |  |  |
| Protein animal source three times a week |  |  |  |  |
|  | Yes | 14 | 10.7 | < 0.0001 |
|  | No | 117 | 89.3 |  |
|  |  |  |  |  |
| Protein animal source four times a week |  |  |  |  |
|  | Yes | 20 | 15.3 | < 0.0001 |
|  | No | 111 | 84.7 |  |
|  |  |  |  |  |
| Protein animal source five times a week |  |  |  |  |
|  | Yes | 13 | 9.9 | < 0.0001 |
|  | No | 118 | 90.1 |  |
|  |  |  |  |  |
| Protein animal source six to seven times a week |  |  |  |  |
|  | Yes | 13 | 9.9 | < 0.0001 |
|  | No | 118 | 90.1 |  |
|  |  |  |  |  |
| Never uses peas and mixed beans |  |  |  |  |
|  | Yes | 12 | 9.2 | < 0.0001 |
|  | No | 119 | 90.8 |  |
|  |  |  |  |  |
| Uses peas and mixed beans once a week |  |  |  |  |
|  | Yes | 20 | 15.3 | < 0.0001 |
|  | No | 111 | 84.7 |  |
|  |  |  |  |  |
| Uses peas and mixed beans twice a week |  |  |  |  |
|  | Yes | 19 | 14.5 | < 0.0001 |
|  | No | 112 | 85.5 |  |
|  |  |  |  |  |
| Uses peas and mixed beans four times a week |  |  |  |  |
|  | Yes | 20 | 15.3 | < 0.0001 |
|  | No | 111 | 84.7 |  |
|  |  |  |  |  |
| Uses peas and mixed beans five times a week |  |  |  |  |
|  | Yes | 32 | 24.4 | < 0.0001 |
|  | No | 99 | 75.6 |  |
|  |  |  |  |  |
| Uses peas and mixed beans six to seven times a week |  |  |  |  |
|  | Yes | 11 | 8.4 | < 0.0001 |
|  | No | 120 | 91.6 |  |
|  |  |  |  |  |
| Has never taken lipids |  |  |  |  |
|  | Yes | 17 | 13 | < 0.0001 |
|  | No | 114 | 87 |  |
|  |  |  |  |  |
| Takes lipids once a week |  |  |  |  |
|  | Yes | 33 | 25.2 | < 0.0001 |
|  | No | 98 | 74.8 |  |
|  |  |  |  |  |
| Takes lipids twice a week |  |  |  |  |
|  | Yes | 26 | 19.8 | < 0.0001 |
|  | No | 105 | 80.2 |  |
|  |  |  |  |  |
| Takes lipids three times a week |  |  |  |  |
|  | Yes | 16 | 12.2 | < 0.0001 |
|  | No | 115 | 87.8 |  |
|  |  |  |  |  |
| Takes lipids four times a week |  |  |  |  |
|  | Yes | 20 | 15.3 | < 0.0001 |
|  | No | 111 | 84.7 |  |
|  |  |  |  |  |
| Takes lipids five times a week |  |  |  |  |
|  | Yes | 15 | 11.5 | < 0.0001 |
|  | No | 116 | 88.5 |  |
|  |  |  |  |  |
| Takes lipids six to seven times a week |  |  |  |  |
|  | Yes | 10 | 7.6 | < 0.0001 |
|  | No | 121 | 92.4 |  |
|  |  |  |  |  |
| Never takes milk and milk products |  |  |  |  |
|  | Yes | 13 | 9.9 | < 0.0001 |
|  | No | 118 | 90.1 |  |
|  |  |  |  |  |
| Takes milk and milk products once a week |  |  |  |  |
|  | Yes | 31 | 23.7 | < 0.0001 |
|  | No | 100 | 76.3 |  |
|  |  |  |  |  |
| Takes milk and milk products twice a week |  |  |  |  |
|  | Yes | 26 | 19.8 | < 0.0001 |
|  | No | 105 | 80.2 |  |
|  |  |  |  |  |
| Takes milk and milk products three times a week |  |  |  |  |
|  | Yes | 20 | 15.3 | < 0.0001 |
|  | No | 111 | 84.7 |  |
|  |  |  |  |  |
| Takes milk and milk products four times a week |  |  |  |  |
|  | Yes | 19 | 14.5 | < 0.0001 |
|  | No | 112 | 85.5 |  |
|  |  |  |  |  |
| Takes milk and milk products five times a week |  |  |  |  |
|  | Yes | 19 | 14.5 | < 0.0001 |
|  | No | 112 | 85.5 |  |
|  |  |  |  |  |
| Takes milk and milk products six to seven times a week |  |  |  |  |
|  | Yes | 7 | 5.3 | < 0.0001 |
|  | No | 124 | 94.7 |  |
|  |  |  |  |  |
| Never takes vitamins and minerals |  |  |  |  |
|  | Yes | 13 | 9.9 | < 0.0001 |
|  | No | 118 | 90.1 |  |
|  |  |  |  |  |
| Takes vitamins and minerals once a week |  |  |  |  |
|  | Yes | 16 | 12.2 | < 0.0001 |
|  | No | 115 | 87.8 |  |
|  |  |  |  |  |
| Takes vitamins and minerals twice a week |  |  |  |  |
|  | Yes | 21 | 16 | < 0.0001 |
|  | No | 110 | 84 |  |
|  |  |  |  |  |
| Takes vitamins and minerals three times a week |  |  |  |  |
|  | Yes | 22 | 16.8 | < 0.0001 |
|  | No | 109 | 83.2 |  |
|  |  |  |  |  |
| Takes vitamins and minerals four times a week |  |  |  |  |
|  | Yes | 22 | 16.8 | < 0.0001 |
|  | No | 109 | 83.2 |  |
|  |  |  |  |  |
| Takes vitamins and minerals five times a week |  |  |  |  |
|  | Yes | 21 | 16 | < 0.0001 |
|  | No | 110 | 84 |  |
|  |  |  |  |  |
| Takes vitamins and minerals six to seven times a week |  |  |  |  |
|  | Yes | 8 | 6.1 | < 0.0001 |
|  | No | 123 | 93.9 |  |
|  |  |  |  |  |
| Nutritional status in terms of MUAC |  |  |  |  |
|  | Normal | 79 | 60.3 | < 0.0001 |
|  | MAM | 30 | 22.9 |  |
|  | SAM | 22 | 16.8 |  |
|  |  |  |  |  |
| Chemotherapy |  |  |  |  |
|  | Yes | 122 | 93.1 | < 0.0001 |
|  | No | 8 | 6.1 |  |
|  | Unknown | 1 | 0.8 |  |
|  |  |  |  |  |
| Radiotherapy | Yes | 12 | 9.2 | < 0.0001 |
|  | No | 118 | 90.1 |  |
|  | Unknown | 1 | 0.8 |  |
|  |  |  |  |  |
| Cancer types |  |  |  |  |
|  | Acute myeloid lymphoma | 2 | 1.5 | < 0.0001 |
|  | Acute lymphoblastic leukemia | 34 | 26.0 |  |
|  | Acute Promyelocytic Leukemia | 1 | 0.8 |  |
|  | Brain tumor | 1 | 0.8 |  |
|  | Burkitt’s Lymphoma | 9 | 6.9 |  |
|  | Ewing sarcoma | 2 | 1.5 |  |
|  | Germ cell tumor | 2 | 1.5 |  |
|  | Hepatoblastoma | 2 | 1.5 |  |
|  | Hodgkin lymphoma | 10 | 7.6 |  |
|  | Nasopharyngeal carcinoma | 1 | 0.8 |  |
|  | Neuroblastoma | 3 | 2.3 |  |
|  | Non-Hodgkin lymphoma | 2 | 1.5 |  |
|  | Osteosarcoma | 1 | 0.8 |  |
|  | Retinoblastoma | 17 | 13.0 |  |
|  | Unknown | 1 | 0.8 |  |
|  | Wilms tumor | 42 | 32.1 |  |
|  | Xerodema | 1 | 0.8 |  |
|  |  |  |  |  |
| Stage of cancer during visit to hospital |  |  |  |  |
|  | Stage 1 | 9 | 17.6 | < 0.0001 |
|  | Stage 2 | 25 | 19.1 |  |
|  | Stage 3 | 45 | 34.4 |  |
|  | Stage 4 | 25 | 19.1 |  |
|  | End of life (palliative) | 23 | 17.6 |  |
|  | Unknown | 4 | 3.1 |  |
|  |  |  |  |  |

**Supplementary Table 2 (ST2):** Food Composition of Frequently Taken Foods by the study population

|  | MACRONUTRIENTS | | | | | | | VITAMINS | | | | | | | MINERALS | | | | | | | |
| --- | --- | --- | --- | --- | --- | --- | --- | --- | --- | --- | --- | --- | --- | --- | --- | --- | --- | --- | --- | --- | --- | --- |
|  | Energy in Kcal (ENERC_KCAL) | Protein in grams (PROCNT) | Animal protein (A_PROTEIN) | Fat in grams (FAT) | Carbo  Hydrates (CHOCDF) | Sugar (SUCS) | Fiber (FIB) | Vit A | Vit A animal | Vit D | Vit E | Vit C | Folate | B Complex (write what is missing) | Ca | P | Mg | K | Na | Fe | Zn | Cu |
| Banana | 116 | 0.8 | 0.0 | 0.2 | 31.2 | 3.3 | 2.3 | 91.0 | 0.0 | 0.0 | 0.0 | 11.0 | 26.0 | Except B12 | 2.0 | 28.0 | 32.0 | 465.0 | 5.0 | 0.6 | 0.1 | 0.1 |
| Beans | 166 | 14 | 0 | 0.7 | 29.9 | 1.3 | 8.4 | 0 | 0 | 0 | 0 | 1 | 170 | B12 | 37 | 186 | 59 | 528 | 3 | 3.8 | 1.4 | 0.3 |
| Cassava | 314 | 26 | 0 | 0.7 | 76.6 | 2.2 | 3.6 | 14 | 0 | 0 | 0 | 72 | 36 | B12 | 46 | 168 | 24 | 583 | 5 | 1.9 | 0.7 | 0.1 |
| Chicken | 200 | 18.8 | 18.8 | 13.2 | 0 | 0 | 0 | 27 | 27 | 0 | 0 | 0 | 4 | - | 9 | 126 | 14 | 127 | 51 | 1 | 1.3 | 0.1 |
| Eggs | 278 | 19.2 | 19.2 | 20.7 | 2.3 | 0 | 0 | 810 | 810 | 8 | 2 | 0 | 12 | - | 96 | 330 | 26 | 333 | 219 | 5,9 | 2.1 | 0.1 |
| Fish | 368 | 78.6 | 58.6 | 3.2 | 0 | 0 | 0 | 49 | 49 | 4 | 4 | 4 | 42 | - | 49 | 483 | 147 | 854 | 273 | 1.8 | 2.1 | 0.1 |
| Fruits | 45 | 0.7 | 0 | 2.2 | 6.6 | 1.8 | 1.9 | 29.1 | 0 | 0 | 0.4 | 26.1 | 23.4 | N,B6,B5,B12 | 16.6 | 11.2 | 10.4 | 190.1 | 1.9 | 0.2 | 0.0 | 0.1 |
| Juice | 45 | 0.7 | 0 | 2.2 | 6.6 | 1.8 | 1.9 | 29.1 | 0 | 0 | 0.4 | 26.1 | 23.4 | N,B6,B5,B12 | 16.6 | 11.2 | 10.4 | 190.1 | 1.9 | 0.2 | 0.0 | 0.1 |
| Kachori | 237 | 6.1 | 0 | 11.4 | 28.8 | 2.4 | 3.4 | 0.4 | 0 | 0 | 0.3 | 11 | 0.2 | Ribof &B12 | 39.7 | 148.8 | 50.4 | 494.7 | 86 | 2.2 | 1,2 | 0.4 |
| Maize | 123.8 | 2.7 | 0 | 1.2 | 25.6 | 0.2 | 2.4 | 0 | 0 | 0 | 0.3 | 0 | 11.6 | B12 | 2 | 81.2 | 43.4 | 98.1 | 11.9 | 1.2 | 0.6 | 0.1 |
| Meat | 168.6 | 12.6 | 13.3 | 24.0 | 0 | 0 | 0 | 13.9 | 0 | 0 | 0 | 3.8 | 7.5 | - | 5.8 | 1o4.36 | 14.6 | 225.7 | 28 | 0.9 | 2.1 | 0.1 |
| Milk | 61 | 3.5 | 3.5 | 3.3 | 4.7 | 4.7 | 0 | 27 | 27 | 0 | 0.1 | 0.5 | 7 | Thiam,b6 | 121 | 95 | 12 | 155 | 0 | 0 | 0 | 0 |
| Okra | 74.3 | 0.8 | 0.1 | 0.1 | 7 | 1.2 | 3.2 | 217.7 | 0 | 0 | 0.3 | 27.5 | 17 | Thia, Ribof, b12 | 15.1 | 22.5 | 10.9 | 771.6 | 107.2 | 0.5 | 0.1 | 0.2 |
| Pancake | 291.4 | 7 | 1.7 | 12.1 | 383 | 0.2 | 1.4 | 38 | 38 | 0.2 | 0.6 | 0 | 20.8 | - | 10.3 | 106.4 | 18.7 | 96.6 | 155.2 | 1 | o.7 | 0.1 |
| Porridge | 382.2 | 13,8 | 0 | 15.6 | 56.1 | 1.8 | 7 | 2.3 | 0 | 0 | 1.6 | 0.3 | 39 | B12 | 11.9 | 306.8 | 103.2 | 513.1 | 15 | 4.6 | 2 | 0.6 |
| Potatoes | 93 | 2 | 0 | 0.1 | 26.6 | 0.2 | 1.5 | 0 | 0 | 0 | 6.7 | 18.6 | 75 | B12 | 5 | 50 | 25 | 39 | 5 | 0.4 | 0.3 | 0.2 |
| Rice | 174 | 2.6 | 0 | 7.4 | 24.6 | 0.1 | 1.3 | 87 | 0 | 0 | 0 | 19 | 16 | - | 7 | 50 | 20 | 242 | 9 | 0.6 | 1.1 | 0.1 |
| Sam | 280 | 11.6 | 7,5 | 11.8 | 30.5 | 0.2 | 1.1 | 0 | 0 | 0 | 0 | 0 | 9 | - | 7 | 101 | 16 | 145 | 17 | 1 | 0.5 | 1.5 |
| Sardine | 112 | 21.4 | 21.4 | 2.3 | 0 | 0 | 0 | 43 | 0 | 12 | 1 | 0 | 11 | - | 48 | 228 | 86 | 461 | 55 | 0.9 | 0.4 | 0 |
| Spar | 371 | 10.5 | 0 | 1 | 77.8 | 0.4 | 2.8 | 0 | 0 | 0 | 0 | 0 | 0 | 0 | 15 | 110 | 22 | 109 | 2 | 1.2 | 0.1 | 0.1 |
| Vegetables | 86.1 | 10 | 0 | 0.2 | 2.1 | 0 | 2 | 192.1 | 0 | 0 | 1.3 | 15.8 | 46.3 | Thiam,ribofBb5,B12 | 35 | 19.5 | 7 | 96 | 7.5 | 23.5 | 0.1 | 0 |
| Yam | 97 | 2.1 | 0 | 0.1 | 22.9 | 0.4 | 1.2 | 0 | 0 | 0 | 0.5 | 12 | 16 | B12 | 8 | 39 | 26 | 3-03 | 3 | 8 | 0.5 | 0.17 |
